# The Bench Press Grip Width Does Not Affect the Number of Repetitions Performed at Different Velocity Loss Thresholds

**DOI:** 10.3390/ijerph18031057

**Published:** 2021-01-25

**Authors:** Alejandro Pérez-Castilla, Ivan Jukic, G. Gregory Haff, Amador García-Ramos

**Affiliations:** 1 Department of Physical Education and Sport, Faculty of Sport Sciences, University of Granada, 18010 Granada, Spain; amagr@ugr.es; 2 Sport Performance Research Institute New Zealand (SPRINZ), Auckland University of Technology, Auckland 1142, New Zealand; ivan.jukic@aut.ac.nz; 3 School of Medical and Health Sciences, Edith Cowan University, Joondalup, WA 6065, Australia; g.haff@ecu.edu.au; 4 Directorate of Psychology and Sport, University of Salford, Salford M5 4WT, Greater Manchester, UK; 5 Department of Sports Sciences and Physical Conditioning, Faculty of Education, Universidad Católica de la Santísima Concepción, Concepción 2850, Chile

**Keywords:** fatigue, resistance training, training prescription, training volume, velocity-based training

## Abstract

This study aimed (I) to compare the number of repetitions that can be completed to failure (XRM) and before reaching a 15%, 30%, or 45% velocity loss threshold (XVLT) in the bench press exercise performed using different grip widths, and (II) to examine the inter-individual variability in the percentage of completed repetitions with respect to the XRM when the set volume is prescribed based on a fixed number of repetitions (FNR) and several velocity loss thresholds (VLT). Nineteen men performed four separate sessions in a random order where there was a single set of repetitions completed to failure against 75% of the one-repetition maximum during the Smith machine bench press exercise using a narrow, medium, wide, or self-selected grip widths. The XRM (*p* = 0.545) and XVLTs (*p* ≥ 0.682) were not significantly affected by grip width. A high and comparable inter-individual variability in the percentage of completed repetitions with respect to the XRM was observed when using both an FNR (median CV = 24.3%) and VLTs (median CV = 23.5%). These results indicate that Smith machine bench press training volume is not influenced by the grip width and that VLTs do not allow a more homogeneous prescription of the set volume with respect to the XRM than the traditional FNR.

## 1. Introduction

Training volume is one of the most critical variables when designing resistance training programs because it affects the resulting neural and morphological training adaptations (i.e., muscular endurance, hypertrophy, maximal strength, or power) [1,2]. The volume (number) of repetitions in a resistance training set has been traditionally prescribed based upon the one-repetition maximum (1RM) or the maximum number of repetitions that can be completed before reaching muscular failure (XRM) [1,2,3]. For example, athletes are instructed to perform a fixed number of repetitions (FNR) against a given relative load (e.g., 5 repetitions at 70% of 1RM) or considering the XRM (e.g., 3 repetitions at the 6RM load). However, performing a FNR against the same %1RM may induce different internal responses among individuals while the use of XRM loads may cause excessive fatigue [4]. An alternative approach to prescribe the training volume consists of recording the velocity at which the repetitions are performed [5,6]. While there are a number of ways to prescribe training volume using repetition velocity, the most commonly used approach involves termination of the set as soon as a pre-determined velocity loss threshold (VLT) has been reached [7,8,9]. For example, athletes are instructed to perform repetitions until a 20% VLT is reached. Recently, it has been postulated that VLTs allow for a greater control of the inter-individual variation in perceptual, metabolic, and neuromuscular responses to resistance training than more traditional methods of prescribing resistance training volume [10,11].

The bench press (BP) exercise is commonly used within resistance training programs for the development of upper-body strength and power both in athletes and in various clinical and elderly populations [12,13,14,15]. One of the frequently overlooked aspects when performing the BP exercise is the impact of grip width on the performance of the exercise [16]. Previous studies have explored the effects of different grip widths on specific muscle activation patterns [17,18], 1RM performance [19,20,21], or kinetic and kinematic outputs [22,23,24]. However, little evidence exists regarding the effects of the grip width on training volume. For instance, Wilk et al. [25] compared the XRM during the free-weight BP exercise using narrow and wide grip widths against 75% of the 1RM. While the authors found no differences in XRM between the grip widths, it is important to note that the loads were prescribed based upon the 1RM achieved with each grip width. Furthermore, although VLTs are now increasingly used to prescribe resistance training volume [7,8,9], it is currently unknown whether the number of repetitions that can be completed before reaching a predetermined VLT (XVLT) is affected by the grip width.

It is well documented that a large inter-individual variability (CV ≥ 15%) exists for the XRM in the Smith machine BP exercise performed against a range of relative loads (50-85% 1RM) [11,26]. Several studies have tried to identify the sources of this inter-individual variability [11,27,28,29]. In that regard, relative BP strength (i.e., the 1RM relative to the individual’s body mass) has been shown to be a poor predictor of the variability between the %1RM and the XRM using either a free-weight [27] or a Smith machine [11], while research on the influence of anthropometric characteristics (e.g., body mass, total arm length, biacromial width, or chest girth) on the XRM is scarce and presents inconclusive evidence [11,28,29].

When looking at the Smith machine BP, the percentage of completed repetitions with respect to the XRM before exceeding certain VLTs presents a lower inter-individual variability than the XRMs (CV = 8.9% vs. 20.1%) [11]. However, more recently, García-Ramos et al. [30] reported a considerable amount of inter-individual variability for the percentage of completed repetitions with respect to the XRM before exceeding a predetermined VLT in the Smith machine BP exercise (CV = 18.8%). These conflicting findings highlight the need for further research on this topic. An argument for using VLTs could be made if a lower inter-individual variability in the percentage of completed repetitions with respect to the XRM exists for the XVLT in comparison to prescribing an FNR. However, no known study has compared the inter-individual variability in the percentage of completed repetitions with respect to the XRM between both approaches of prescribing resistance training volume (FNR vs. VLT).

To address these gaps in the literature, subjects in the present study performed, on separate occasions, single sets of repetitions to failure against the 75% 1RM load in the Smith machine BP exercise using four different grip widths (narrow, medium, wide, and self-selected). The current study aimed to examine: (I) the effects of the grip width on XRM and XVTL (15%, 30%, and 45%) as well as slowest (MV_slowest_) and fastest (MV_fastest_) repetition in the set; (II) the association between different grip widths for XRM and XVLT, as well as between XRM and XVLT separately for each grip width; (III) the effects of relative strength and anthropometric characteristics on XRM and XVLT; and (IV) the inter-individual variability in the percentage of completed repetitions with respect to the XRM when the number of repetitions is prescribed based on FNR and VLT. Based on the findings of previous studies [11,25,27,30], we hypothesized that: (I) the XRM, XVTL, as well as MV_slowest_ and MV_fastest_ in the set would not be affected by the grip width; (II) significant correlations would be detected between the different grip widths for XRM and XVLTs, as well as between XRM and XVLTs for each grip width; (III) neither the relative strength nor the anthropometric characteristics would be significantly correlated with the XRM or XVLT; and (IV) a lower inter-individual variability in the percentage of completed repetitions with respect to the XRM would be observed using VLTs compared to a FNR.

## 2. Materials and Methods

### 2.1. Subjects

Nineteen male resistance-trained sports science students volunteered to participate in this study (Table 1). Inclusion criteria for the subjects were: (i) having at least two years of resistance training experience (2–5 sessions per week) with the goal of developing muscular force; (ii) having a relative 1RM strength higher than 0.70 in the BP exercise; and (iii) being free from any physical limitations that could compromise the study procedures. The sample size was similar to that considered in previous studies that also examined the acute effects of different VTLs (*n* = 16 to 20) [10,31] and an a priori power analysis was not performed due to the multiple statistical analyses performed. All subjects were informed of the study procedures and signed a written informed consent form before the commencement of the study. The study protocol adhered to the tenets of the Declaration of Helsinki and was approved by the institutional review board (IRB approval: 491/CEIH/2018).

### 2.2. Study Design

A crossover design was used to examine the effects of different grip widths on the XRM and XVLT during the BP exercise performed in a Smith machine (GervaSport, Madrid, Spain). Subjects came to the laboratory on five occasions separated by 48–72 h. The first session was used to determine subjects’ anthropometric characteristics and the BP 1RM. Sessions 2–5 consisted of performing a single set of repetitions to failure against the 75% 1RM load. A single BP grip width (narrow, medium, wide, or self-selected) was used in each session in a random order. Subjects were instructed to avoid any strenuous physical activity over the course of the study. All testing sessions were performed at the same time of the day for each subject (±1 h) and under similar environmental conditions (∼22 °C and ∼60% humidity).

### 2.3. Procedures

#### 2.3.1. Anthropometric Measures and 1RM Assessment (Session 1)

Body height, body mass, biacromial width (measured as the distance between the left and right acromioclavicular joints), mesosternal perimeter (measured as the contour of the thorax at the level of the mesosternal point), anteroposterior chest diameter (measured as the distance between the mesosternal point and the spinous process located at that level), transverse chest diameter (measured as the distance between the most lateral points of the thorax at the level of the mesosternal point), and total arm length (measured as the average distance of both arms from the acromioclavicular joint to the ulna’s styloid process) were measured at the beginning of the session following the protocol of the International Society for the Advancement of Kinanthropometry [32].

Following the anthropometric assessment, a standard incremental loading test was used to determine the Smith Machine BP 1RM using the narrow grip width [33]. The warm-up consisted of jogging, dynamic stretching, upper-body joint-mobilization exercises, and 1 set of 5 repetitions with an external load of 15 kg. Thereafter, the external load was progressively increased in 10 kg increments until the mean velocity was lower than 0.50 m·s^−1^. From that moment, the load was increased from 5 to 1 kg until the 1RM load was reached. The rest between sets was set to 4 min, and 1–2 repetitions were performed with each load.

#### 2.3.2. Repetitions-to-Failure Tests (Session 2–5)

Each session consisted of a single set of repetitions to failure against the 75% 1RM load. Only one grip width was tested in each session and the same absolute load was used in all sessions. All sessions began with the same general warm-up described for session 1. The specific warm-up included the specific grip width of the session with subjects performing 1 set of 10, 5, and 3 repetitions at 30%, 50%, 70% of 1RM, respectively, followed by 1 repetition at 90% of 1RM. The set of repetitions to failure ended when the subjects: (i) were unable to complete a repetition with the full range of motion; or (ii) paused for more than one second with the arms in the extended position [34]. Subjects were instructed to perform as many repetitions as possible and velocity performance feedback was verbally provided after each repetition to encourage them to perform all repetitions at the maximal intended velocity.

#### 2.3.3. Description of the BP Exercise

The BP was performed according to the standard five-point body contact position technique (head, upper back, and buttocks placed firmly on the bench with both feet flat on the floor). Subjects started the task lying supine on a flat bench, with their feet resting on the floor, their elbows fully extended, and their hands placed on the bar using either a narrow, medium, wide, or self-selected grip width. From this position, they lowered the bar in a controlled manner until it made contact with the chest, held this position for approximately two seconds, and then lifted the bar as fast as possible until their elbows reached full extension [23,24]. The position of the bench was adjusted so that the vertical projection of the bar corresponded to each subject’s intermammary line. The distance between the index fingers was recorded and marked on the bar with a tape and kept constant for each subject throughout all lifts [21]. The narrow grip width represented a 100% of the biacromial width (38.6 ± 2.6 cm (35–45 cm)), the medium grip width a 150% of the biacromial width (57.9 ± 3.8 cm (52.5–67.5 cm)), the wide grip width a 200% of the biacromial width (77.3 ± 5.1 cm (70–90 cm)), and the self-selected grip width a 173 ± 22% of the biacromial width (66.7 ± 8.7 cm (44–78 cm)) (mean ± standard deviation (range)).

#### 2.3.4. Equipment

Anthropometric measurements were performed by means of a steel flexible tape (Rosscraft Anthrotape; Rosscraft Innovations Inc., Vancouver, Canada) and a large sliding calliper (Campbell 20; Rosscraft Innovation Inc., Vancouver, Canada). Body height was measured using a wall-mounted stadiometer (Seca 202; Seca Ltd., Hamburg, Germany), while the body mass was assessed using a contact electrode foot-to-foot body fat analyzer system (TBF-300A; Tanita Corporation of America Inc., Arlington Heights, IL, USA). The BP exercise was performed in a Smith machine (GervaSport, Madrid, Spain). A linear velocity transducer (T-Force System; Ergotech, Murcia, Spain) was used to collect the mean velocity of all repetitions. The T-Force System interfaced to a personal computer by means of a 14-bit resolution analog-to-digital data acquisition board and custom software. Instantaneous velocity was sampled at a frequency of 1000 Hz and subsequently smoothed with a 4th order low-pass Butterworth digital filter with no phase shift and 10 Hz cut-off frequency. Validity and reliability of the T-Force system for the recording of mean velocity during the BP exercise has been reported elsewhere [35].

### 2.4. Statistical Analyses

Descriptive data are presented as mean ± SDs, while the coefficient of variation (CV) and Spearman’s rho correlation coefficients (*r_s_*) are indicated as the median value and range. The Shapiro–Wilk test revealed a violation of the normal distribution assumption for some variables (*p* < 0.05). Consequently, the Friedman test was used to compare the XRM and XVLTs as well as MV_slowest_ and MV_fastest_ in the set between the four BP grip widths. The *r_s_* was used to quantify the associations between: (I) the different grips widths for XRM and XVLTs; (II) the XRM and XVLTs for each grip width; and (III) the relative strength and anthropometric characteristics with the XRM and XVLT for each grip width. Finally, the inter-individual variability in the percentage of completed repetitions with respect to the XRM was calculated when performing a fixed number of repetitions (FNR of 5, 8, and 10) or when reaching certain velocity loss thresholds (VLT of 15%, 30%, and 45%) (Equation (1)). The FNR of 5, 8, and 10 were selected based on the average number of repetitions completed by the subjects when reaching the 15%, 30%, and 45% VLT, respectively. Note that the velocity loss limit was determined from MV_fastest_ within the set until the threshold was exceeded for the first time (e.g., the set would stop below 0.59 m·s^−1^ for a MV_fastest_ of 0.69 m·s^−1^ and a VTL of 15%). Statistical analyses were performed using the software package SPSS (IBM SPSS version 25.0, Chicago, IL, USA). Statistical significance was set at *p* < 0.05.

(1) $$\mathrm{CV}\;(\%)\;=\;\frac{Between-subjects\;SD}{Subjects’\;mean\;score}\;\times \;100$$

## 3. Results

Friedman tests did not reveal a significant effect for XRM (*χ*^2^_(3, N = 21)_ = 2.13, *p* = 0.545), XVLT-15% (*χ*^2^_(3, N = 21)_ = 1.14, *p* = 0.768), XVLT-30% (*χ*^2^_(3, N = 21)_ = 1.50, *p* = 0.682), or XVLT-45% (*χ*^2^_(3, N = 21)_ = 0.79, *p* = 0.853), MV_fastest_ (*χ*^2^_(3, N = 21)_ = 0.54, *p* = 0.909), or MV_slowest_ (*χ*^2^_(3, N = 21)_ = 0.68, *p* = 0.879) (Table 2). MV_fastest_ was always observed among the first four repetitions (1st repetition = 63%, 2nd repetition = 28%, 3rd repetition = 8%, 4th repetition = 1%), while MV_slowest_ was always attained during the last repetition. Subjects completed repetitions faster than the VLTs once the thresholds were exceeded for the first time in 13 out of 76 occasions for the 15% VLT (+1 repetitions = 6 occasions, +2 repetitions = 4 occasions, +3 repetitions = 1 occasion, +4 repetitions = 2 occasions), in 4 out of 76 occasions for the 30% VLT (+1 repetitions = 4 occasions), and 4 out of 76 occasions for the 45% VLT (+1 repetitions = 3 occasions, +2 repetitions = 1 occasion).

There were positive correlations between the grip widths (*r_s_* = 0.823 (0.795–0.898)) for both XRM and XVLTs (Table 3). In addition, regardless of the grip width, the XRM and XVLTs were also positively correlated (*r_s_* = 0.623 (0.380–0.902)) (Table 4).

The relative strength was negatively correlated with the XRM (*r_s_* = −0.697), XVLT-30% (*r_s_* = −0.533), and XVLT-45% (*r_s_* = −0.600) during the BP performed with a medium grip width. Body height was positively correlated with the XRM (*r_s_* = 0.587), XVLT-30% (*r_s_* = 0.515), and XVLT-45% (*r_s_* = 0.546) during the BP performed with a medium grip width. Furthermore, the total arm length was positively correlated with the XRM (*r_s_* = 0.522) during the BP performed with a medium grip width and with the XVLT-15% (*r_s_* = 0.487) during the BP performed with a wide grip width. No significant correlations were found for the remaining 120 comparisons (Table 5).

**Figure 1:**
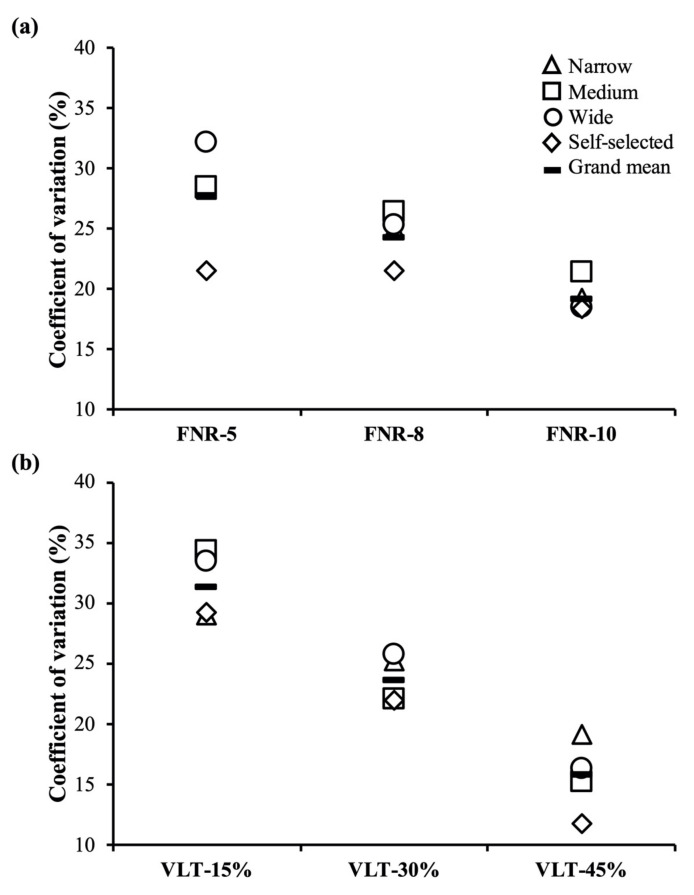
Coefficients of variation for the percentage of completed repetitions with respect to the maximal number of repetitions performed to failure when reaching a fixed number of repetitions ((**a**) FNR; upper panel) or a given velocity loss threshold ((**b**) VLT; lower panel) for each bench press grip width. The grand mean was calculated as the average value of the four bench press grip widths. The selected FNR were based on the average number of repetitions completed by the participants when reaching the VLT.

A high and comparable inter-individual variability in the percentage of completed repetitions with respect to the XRM were observed when using both a FNR (CV = 24.3% (19.2%–27.7%)) and VLTs (CV = 23.5% (15.8%–31.3%)) (Table 6). Of special note is that the inter-individual variability in the percentage of completed repetitions with respect to the XRM always decreased with the increment in the number of repetitions (Figure 1).

## 4. Discussion

This study was designed to examine the effects of different grip widths on the XRM and XVLT during the Smith machine BP exercise. The main findings of this study were that: (I) XRM, XVLTs, MV_slowest_, and MV_fastest_ were not significantly affected by the grip width; (II) there were positive correlations between the grip widths for both XRM and XVLTs and between XRM and XVLTs across the grip widths; (III) relative strength and anthropometric characteristics did not consistently present a significant correlation with the XRM or XVLT; and (IV) a high and comparable inter-individual variability was observed using both FNR and VTL. When considered collectively, these results suggest that the training volume is not influenced by Smith machine BP grip width and that using VLTs do not allow for a more homogeneous prescription of the set volume with respect to the XRM when compared to using the traditional FNR methodology.

Supporting our first hypothesis, the Smith machine BP grip width did not affect the XRM and XVTL completed against the same absolute load (75% of the narrow grip width 1RM). These findings are in line with those previously demonstrated by Wilk et al. [25], who did not find significant differences between the narrow (95% of the biacromial width) and wide (200% of the biacromial width) grip widths for the XRM completed against the grip-specific 75% 1RM loads during the free-weight BP exercise. It has previously been suggested that changes in BP grip width can affect 1RM performance [19,20,21] and kinetic and kinematic outputs [22,23,24]. However, based on the findings by Wilk et al. [25], and the results of the present study, it seems that the grip width might not affect the total repetition volume or the repetition volume before exceeding a pre-determined VLT. Even more important is the fact that the equipment (machine-based vs. free-weight movement) used to perform the BP exercise does not appear to influence the reported findings, but caution should be taken due to the methodological differences between the studies. Furthermore, García-Ramos et al. [30] has recently shown that MV_fastest_ was predominantly observed during the 1st repetition (53%) and 2nd repetition (32%) and that individuals sometimes produced a velocity output above a VLT once this threshold is exceeded for the first time (on 0 to 4 occasions for 15% and 30% VLTs, and on 0 to 2 occasions for 45% VLT). In the present study, and in agreement with the findings by García-Ramos et al. [30], MV_fastest_ was also predominantly observed during the 1st repetition (63%) and 2nd repetition (28%). In addition, subjects in the present study frequently produced velocity outputs above 15% (13 occasions), 30% (4 occasions), and 45% (4 occasions) VLTs once these thresholds were exceeded for the first time. It is also worth noting that the MV_slowest_ was always observed in the last repetition of the set to failure. In that regard, the present study provides additional evidence on the importance of considering the reference repetition when implementing VLTs to prescribe and monitor training volume during resistance training. Collectively, these findings suggest that XRM, VLTs, MV_slowest_, and MV_fastest_ are not affected by the grip width during the Smith machine BP exercise. Therefore, the self-selected grip width could be the simplest and most ecologically valid strategy of Smith machine BP execution during resistance training.

Previous studies have explored the influence of relative strength and certain anthropometric characteristics on XRM [11,27,28,29], but no previous study has looked at the effects of these factors on XVLTs. Supporting our third hypothesis, neither the relative strength nor the anthropometric characteristics were systematically related to the XRM and XVLTs. These results are in line with previous studies that failed to find significant relationships between XRMs and relative strength in the BP exercise against a range of relative loads (50–90% 1RM) [11,27]. Our results are also in agreement with previous studies that did not find a clear relationship between the XRM and anthropometric characteristics such as body height (*r* range = −0.50 to 0.16), body mass (*r* range = −0.44 to 0.21), or total arm length (*r* range = −0.46 to 0.16) against a range of relative loads (40–85% 1RM) [11,28]. However, our results are in disagreement with other investigations that have reported high correlations between the XRM and biacromial width (*r* range = −0.60 to −0.50) and XRM and chest girth (*r* range = 0.56 to 0.60) against different relative loads (40–100% 1RM) [28,29]. The discrepancy between the findings could likely be explained by the differences in study methodologies, technical execution of the BP exercise, and the sample size. Nevertheless, it is important to note that the present study expands the current knowledge while examining not only XRM, but also certain XVLTs in the BP exercise with different grip widths. Based on available evidence, the large inter-individual variability observed for the XRM might be mainly caused by other factors such as training background or specific muscle characteristics of the individuals [36] rather than relative strength or anthropometric characteristics.

Resistance training volume has been commonly prescribed using a predetermined FNR to be completed in each exercise set [2]. However, since there is a large inter-individual variability in the XRM completed against a given relative load [11,26], requiring all individuals to perform the same FNR will likely result in a different training stimulus for each athlete. In this regard, one of the greatest challenges facing coaches, strength, and conditioning professionals is how to accurately prescribe training volume to elicit specific adaptations. As a potential solution, previous research has proposed using different VLTs [7,8,9] and VLT-based equations to estimate with a low inter-individual variability (CV = 2.7%–12.1%) the number of repetitions left in reserve in a set during the BP exercise [11,37]. However, our fourth hypothesis was rejected because a high and comparable inter-individual variability in the percentage of completed repetitions with respect to the XRM was observed for both FNR and VLT. These findings are in contrast to a previous study conducted by Gonzalez-Badillo et al. [11], who observed a considerably lower inter-individual variability for the percentage of completed repetitions with respect to the XRM before exceeding a predetermined VLT in the same exercise against the 75% 1RM load (VLT-15%: 31.3% vs. 10.9%; VTL-30%: 23.5% vs. 8.5%; VLT-45%: 15.8% vs. 7.1%). However, our findings are in agreement with a more recent study by García-Ramos et al. [30], which showed a comparable inter-individual variability of the completed repetitions with respect to the XRM before exceeding a predetermined VLT against multiple short (≤12 XRM) or long (>12 XRM) training sets in the Smith machine BP exercise. Such discrepancies could be attributed in part to methodological differences between the studies (e.g., velocity variable (mean velocity vs. mean propulsive velocity) or execution mode (touch-and-go technique vs. concentric-only technique from the bar holders or chest)). Furthermore, in agreement with both González-Badillo et al. [11] and García-Ramos et al. [30], the inter-individual variability in the percentage of completed repetitions with respect to the XRM for both VLT and FNR tended to progressively decrease as the number of repetitions completed increased. Nevertheless, due to the high inter-individual variability in the percentage of the completed repetitions with respect to XRM for both VLT and FNR, caution should be practiced when prescribing resistance training since neither of the strategies guarantee that all individuals are experiencing similar levels of exertion after completing each set of the Smith machine BP exercise.

## 5. Conclusions

The grip width during the Smith machine BP exercise did not affect XRM or XVLTs. In addition, the fastest and the slowest repetitions were also not affected by the grip width during the BP exercise against the same relative load in the set to muscular failure. Therefore, coaches, strength, and conditioning professionals are encouraged to implement the self-selected grip width as the simplest and most ecologically valid strategy of Smith machine BP execution during resistance training. Furthermore, while XRM and XVLTs were not affected by the relative strength or anthropometric characteristics of the individuals, a large inter-individual variability was observed for the percentage of completed repetitions with respect to the XRM, further suggesting that neither FNR nor VLT guarantee that all individuals are experiencing a similar level of exertion after completing each training set in the Smith machine BP exercise. In that regard, individual determination of the XRM and XVLTs is therefore recommended for more accurate and objective monitoring of repetition volume during each set of the BP exercise. However, since our sample size was relatively small and consisted of exclusively males with moderate resistance training experience and variable maximal strength values (BP 1RM = 0.97 ± 0.19 kg⋅body mass^−1^), future studies should investigate whether the results of the present study can be extrapolated to females or athletes with higher resistance training experience.

## Figures and Tables

**Table 1:** Participants’ characteristics.

	Mean ± SD	Range
Age (year)	22.6 ± 3.7	19–33
Body height (m)	1.77 ± 0.06	1.68–1.92
Body mass (kg)	77.4 ± 13.3	63.2–109.8
Bench press 1RM (kg)	74.2 ± 13.3	49–100
Relative bench press 1RM (1RM/body mass)	0.97 ± 0.19	0.74–1.41
Biacromial width (cm)	38.6 ± 2.6	35–45
Mesosternal perimeter (cm)	94.5 ± 9.2	80–115
Anteroposterior chest diameter (cm)	20.6 ± 2.6	16.5–26
Transverse chest diameter (cm)	27.6 ± 2.9	20–32
Total arm length (cm)	59.9 ± 3.5	53–65

SD, standard deviation; 1RM, one-repetition maximum.

**Table 2:** Comparison of the fastest mean velocity (MV_fastest_), slowest mean velocity (MV_slowest_), number of repetitions performed to failure (XRM), and number of repetitions performed before reaching certain velocity loss thresholds (XVLT) between the four bench press grip widths.

Variable	Grip Width				Friedman Test	
	Narrow	Medium	Wide	Self-Selected	*χ* ^2^	*p*-Value
MV_fastest_ (m·s^−1^)	0.56 ± 0.10 [0.39, 0.71]	0.57 ± 0.07 [0.44, 0.68]	0.56 ± 0.08 [0.44, 0.72]	0.57 ± 0.06 [0.48, 0.69]	0.54	0.909
MV_slowest_ (m·s^−1^)	0.16 ± 0.03 [0.09, 0.22]	0.17 ± 0.03 [0.09, 0.23]	0.17 ± 0.03 [0.10, 0.22]	0.17 ± 0.02 [0.12, 0.21]	0.68	0.879
XRM	11.8 ± 3.5 [7, 19]	12.6 ± 3.5 [7, 21]	12.1 ± 3.4 [6, 18]	12.3 ± 2.8 [8, 19]	2.13	0.545
XVLT-15%	4.6 ± 1.9 [2, 8]	4.6 ± 1.7 [2, 8]	4.4 ± 1.8 [2, 8]	4.6 ± 1.9 [2, 11]	1.14	0.768
XVLT-30%	7.6 ± 1.8 [5, 12]	7.8 ± 1.9 [5, 12]	7.4 ± 2.2 [4, 12]	7.9 ± 1.9 [6, 12]	1.50	0.682
XVLT-45%	9.7 ± 2.3 [6, 14]	10.2 ± 2.3 [7, 14]	9.8 ± 2.6 [5, 15]	9.8 ± 2.6 [7, 15]	0.79	0.853

Data are mean ± standard deviation (range). *χ*^2^, Chi-square.

**Table 3:** Correlations between the four bench press grip widths for the number of repetitions performed to failure (XRM) and number of repetitions performed before reaching certain velocity loss thresholds (XVLT).

Variable	Grip Width	Narrow	Medium	Wide
XRM	Medium	0.488 *		
	Wide	0.554 *	0.440	
	Self-selected	0.682 *	0.628 *	0.693 *
XVLT-15%	Medium	−0.182		
	Wide	0.700 *	0.182	
	Self-selected	0.081	0.176	0.310
XVLT-30%	Medium	0.488 *		
	Wide	0.713 *	0.380	
	Self-selected	0.746 *	0.570 *	0.510 *
XVLT-45%	Medium	0.469 *		
	Wide	0.380	0.557 *	
	Self-selected	0.756 *	0.637 *	0.483 *
Grand mean	Medium	0.795 *		
	Wide	0.835 *	0.802 *	
	Self-selected	0.898 *	0.822 *	0.833 *

The grand mean was calculated as the average value of the XRM and XVLTs. *, significant correlation (*p* < 0.05).

**Table 4:** Correlations between the number of repetitions performed to failure (XRM) and the number of repetitions performed before reaching certain velocity loss thresholds (XVLT) separately for each bench press grip width.

Grip Width	Variable	XRM	XVLT-15%	XVLT-30%
Narrow	XVLT-15%	0.549 *		
	XVLT-30%	0.686 *	0.778 *	
	XVLT-45%	0.891 *	0.598 *	0.829 *
Medium	XVLT-15%	0.276		
	XVLT-30%	0.846 *	0.209	
	XVLT-45%	0.937 *	0.279	0.909 *
Wide	XVLT-15%	0.477 *		
	XVLT-30%	0.694 *	0.540 *	
	XVLT-45%	0.897 *	0.431	0.837 *
Self-selected	XVLT-15%	0.290		
	XVLT-30%	0.814 *	0.443	
	XVLT-45%	0.892 *	0.451	0.900 *
Grand mean	XVLT-15%	0.380 *		
	XVLT-30%	0.765 *	0.480 *	
	XVLT-45%	0.902 *	0.433 *	0.865 *

The grand mean was calculated as the average value of the four bench press grip widths. *, significant correlation (*p* < 0.05).

**Table 5:** Correlations of the number of repetitions performed to failure (XRM) and before reaching certain velocity loss thresholds (XVLT) with the relative strength and anthropometric characteristics for each bench press grip width.

Variable	Grip Width	Relative Strength	Body Height	Body Mass	Biacromial Width	Mesosternal Perimeter	Anteroposterior Chest Diameter	Transverse Chest Diameter	Total Arm Length
XRM	Narrow	−0.170	0.155	−0.166	−0.383	−0.257	−0.202	−0.274	0.293
	Medium	−0.697 *	0.587 *	0.077	−0.064	0.094	0.191	−0.179	0.522 *
	Wide	0.103	0.294	−0.131	−0.279	−0.223	−0.235	−0.229	0.278
	Self-selected	−0.244	0.333	−0.091	−0.262	−0.217	−0.038	−0.203	0.301
XVLT-15%	Narrow	−0.301	0.172	−0.048	−0.239	0.044	0.06	−0.217	0.285
	Medium	0.027	0.423	0.330	0.296	0.358	0.341	0.038	0.239
	Wide	−0.180	0.155	−0.084	−0.238	−0.107	0.043	−0.421	0.138
	Self-selected	−0.201	0.354	0.066	−0.206	−0.122	0.013	−0.330	0.487 *
XVLT-30%	Narrow	−0.091	0.279	−0.099	−0.350	0.193	−0.093	−0.209	0.373
	Medium	−0.533 *	0.515 *	0.146	0.078	0.187	0.205	−0.303	0.429
	Wide	0.090	0.271	−0.042	−0.133	−0.070	−0.094	−0.115	0.230
	Self-selected	−0.144	0.419	−0.157	−0.350	−0.219	−0.030	−0.312	0.293
XVLT-45%	Narrow	−0.088	0.137	−0.112	−0.331	−0.241	−0.097	−0.256	0.231
	Medium	−0.600 *	0.546 *	0.112	0.026	0.085	0.185	−0.181	0.454
	Wide	−0.003	0.314	0.021	−0.039	0.004	−0.103	0.003	0.243
	Self-selected	−0.086	0.323	−0.152	−0.335	−0.300	−0.136	−0.331	0.325

The relative strength is calculated as the one-repetition maximum divided by the subject’s body mass. *, significant correlation (*p* < 0.05).

**Table 6:** Percentage of completed repetitions with respect to the maximal number of repetitions performed to failure when reaching a fixed number of repetitions (FNR) or a given velocity loss threshold (VLT) for each bench press grip width.

Variable	Grip Width			
	Narrow	Medium	Wide	Self-Selected
FNR-5	45.8 ± 13.0%	42.7 ± 12.2%	45.1 ± 14.5%	42.7 ± 9.2%
	[26.3%, 71.4%]	[23.8%, 71.4%]	[27.8%, 83.3%]	[26.3%, 62.5%]
VLT-15%	42.5 ± 12.3%	38.4 ± 13.2%	36.8 ± 12.3%	38.1 ± 11.1%
	[11.1%, 62.5%]	[13.3%, 57.1%]	[14.3%, 57.1%]	[16.7%, 57.9%]
FNR-8	71.8 ± 17.9%	67.5 ± 17.8%	69.6 ± 17.7%	68.3 ± 14.8%
	[42.1%, 100%]	[38.1%, 100%]	[44.4%, 100%]	[42.1%, 100%]
VLT-30%	66.5 ± 12.7%	63.0 ± 9.6%	62.3 ± 10.2%	65.3 ± 7.7%
	[38.9%, 87.5%]	[41.2%, 78.6%]	[38.9%, 77.8%]	[50.0%, 77.8%]
FNR-10	83.5 ± 16.5%	80.7 ± 17.3%	82.5 ± 15.3%	83.5 ± 15.4%
	[52.6%, 100%]	[47.6%, 100%]	[55.6%, 100%]	[52.6%, 100%]
VLT-45%	83.9 ± 10.6%	82.5 ± 9.5%	82.0 ± 8.4%	79.8 ± 9.3%
	[61.1%, 100%]	[61.9%, 100%]	[61.1%, 100%]	[58.3%, 100%]

Data are mean ± standard deviation (range). The selected FNR were based on the average number of repetitions completed by the participants when reaching the VLT.
